# Sex Differences in X-ray-Induced Endothelial Damage: Effect of Taurine and N-Acetylcysteine

**DOI:** 10.3390/antiox12010077

**Published:** 2022-12-29

**Authors:** Ilaria Campesi, Antonio Brunetti, Giampiero Capobianco, Adriana Galistu, Andrea Montella, Francesca Ieri, Flavia Franconi

**Affiliations:** 1 Dipartimento di Scienze Biomediche, Università degli Studi di Sassari, 07100 Sassari, Italy; 2 Laboratorio Nazionale Medicina e Farmacologia di Genere, Istituto Nazionale Biostrutture Biosistemi, 07100 Sassari, Italy; 3 Clinica Ostetrica e Ginecologica, Dipartimento di Medicina, Chirurgia e Farmacia, Università degli Studi di Sassari, 07100 Sassari, Italy; 4 Unità Operativa di Genetica e Biologia dello Sviluppo, Azienda Ospedaliero Universitaria di Sassari, 07100 Sassari, Italy; 5 Laboratorio PHYTOLAB (Pharmaceutical, Cosmetic, Food Supplements Technology and Analysis)-DiSIA Università degli Studi di Firenze, 50019 Firenze, Italy

**Keywords:** ionizing radiation, taurine, N-acetycysteine, sex differences, HUVECs

## Abstract

Ionizing radiation (IR) can induce some associated pathological conditions due to numerous cell damages. The influence of sex is scarcely known, and even less known is whether the effect of antioxidants is sex-dependent. Given the increased use of IR, we investigated whether male human umbilical vein endothelial cells (MHUVECs) and female human umbilical vein endothelial cells (FHUVECs) respond differently to IR exposure and whether the antioxidants 10 mM taurine (TAU) and 5 mM N-acetylcysteine (NAC) can prevent IR-induced damage in a sex-dependent way. In untreated cells, sex differences were observed only during autophagy, which was higher in FHUVECs. In non-irradiated cells, preincubation with TAU and NAC did not modify viability, lactate dehydrogenase (LDH) release, migration, or autophagy, whereas only NAC increased malondialdehyde (MDA) levels in FHUVECs. X-ray irradiation increased LDH release and reduced viability and migration in a sex-independent manner. TAU and NAC did not affect viability while reduced LDH release in irradiated cells: they have the same protective effect in FHUVECs, while, TAU was more protective than NAC in male cells.. Moreover, TAU and NAC significantly promoted the closure of wounds in both sexes in irradiated cells, but NAC was more effective at doing this in FHUVECs. In irradiated cells, TAU did not change autophagy, while NAC attenuated the differences between the sexes. Finally, NAC significantly decreased MDA in MHUVECs and increased MDA in FHUVECs. In conclusion, FHUVECs appear to be more susceptible to IR damage, and the effects of the two antioxidants present some sex differences, suggesting the need to study the influence of sex in radiation mitigators.

## 1. Introduction

It is well established that sex is a major determinant in physiology and pathology [1] which influences many cellular processes, including the cellular redox balance [2,3,4,5,6]. However, it is not yet clearly known whether antioxidant activities are influenced by sex, although some phenolic antioxidants [7,8] and glutathione [9,10] display sex-gender-specific activities.

In humans, there has been an increase in ionizing radiation (IR) exposure because of defence sectors, the nuclear power industry, and health care’s use of IR [11]. Importantly, a personalized risk assessment of IR exposure for health professionals and other work sectors, including spaceflight, is still missing [12]. Demographic factors, such as sex, seem to influence IR sensitivity; unfortunately, only a few relevant studies are available [13]. The International Commission on Radiological Protection (ICRP) does not recommend the radiosensitivity of distinct cohorts [13]. Nevertheless, available findings suggested that women have a larger radiosensitivity than men [13]. For example, the risk of solid cancer-induced irradiation is larger in women than in men (about 2-fold), and this risk is independent of the exposure mechanism [14]. IR exposure can induce radiation-associated pathology [13,15,16,17,18,19] through reactive oxygen species (ROS). For example, acute low doses of X-rays increase apoptosis in endothelial cells [20], whereas IR exposure produces an increase in autophagy, which is a process implicated in the elimination of damaged intracellular structures that induces vascular injury and endothelial dysfunction [21]. Importantly, endothelial dysfunction has a role in radiation-induced cardiovascular diseases [19,22,23,24,25]. Despite sex differences, both men and women receive the same protection standard, even though women have more cancers and cardiovascular diseases following nuclear accidents [13].

Damage reduction induced by IR is based on antioxidants [26,27,28] and/or glutathione-elevating compounds [29]. Different natural antioxidants are present in foods, such as taurine (TAU), and they may be beneficial for many diseases characterized by oxidative stress, such as diabetes mellitus [30,31], since the TAU transporter is also inversely linked to retinopathy [32] and blood hypertension [33]. In the human body, TAU is produced by the oxidative catabolism of cysteine or by oxidation of hypotaurine [34]. It also has antioxidant effects due to multiple mitochondrial or non-mitochondrial mechanisms, but it does not act as a classical scavenger of ROS generation [35]. For example, it detoxifies H_2_O_2_, hydroxyl radicals, and nitric oxide, and it is a cytoprotective agent attenuating lipid peroxidation and calcium overload [35]. TAU is an attractive candidate for the prevention of IR-induced damage due to its high safety, although its mechanism is still unclear [28]. In addition, exposure to high doses of IR increases TAU urinary excretion [36], and cancer patients are TAU-depleted after cytotoxic chemotherapy and/or radiation therapy [37]. However, it is not known whether the protective effects of TAU against cellular damage are influenced by sex. However, in some experimental models, such as hereditary cardiomyopathy of the hamster, TAU reduces heart weight only in males, but it decreases early mortality in both sexes [38]. Finally, TAU neuroprotective action versus pilocarpine-induced seizures is present only in males [39].

N-acetylcysteine (NAC) is a synthetic acetyl derivative of cysteine which is clinically used in paracetamol intoxication and as a mucolytic agent [40]. However, its clinical use is expanding as it is also used for renal protection, atrial fibrillation [40,41], and in the treatment of psychiatric and neurological disorders, such as autism, addiction, and depressive disorders, where it has some beneficial effects [40]. It is also present in over-the-counter nutritional supplements as an antioxidant [40] as it can prevent or attenuate ROS-induced damage favouring glutathione formation [29,40]. Recently, a new antioxidant mechanism of NAC was proposed based on its capacity to break thiol proteins generating free thiols, which have larger antioxidant activity than NAC [42]. Overall, NAC use appears to be safe [40].

Thiol NAC reduces IR damage in many tissues and cells [29,43,44,45], and it is also implicated in cell apoptosis and autophagy of endothelial cells [46,47,48,49,50]. In addition, NAC pre-incubation is unable to reduce radiation-induced ICAM-1 expression in irradiated human umbilical venous endothelial cells (HUVECs) [51].

Nevertheless, the majority of glutathione investigations enrolled a single gender, and sexual dimorphisms in glutathione metabolism and glutathione-dependent signaling have been reported [10], which reflects sex differences in human diseases, such as cardiovascular diseases, metabolic disorders, and neurodegenerative diseases, such as Alzheimer’s disease and Parkinson’s disease [10].

The purpose of this work is to understand whether the risks of radiation exposure are sex-dependent in male and female HUVECs (MHUVECs and FHUVECs, respectively), and to understand whether TAU and NAC can prevent IR-induced damage in a sex-dependent way given their high safety profiles and low costs [52,53].

## 2. Methods

### 2.1. Donors

Umbilical cords from healthy human male and female neonates which were vaginally delivered at term (37–42 weeks) at the Obstetrics and Gynecology Clinic at the University of Sassari were selected from healthy, non-obese, and non-smoking mothers who were drug-free, except for folic acid and iron supplementation. HUVECs were obtained only from the umbilical cords of normal-weight neonates, according to Bertino et al. [54] (2430–4050 and 2550–4190 g for males and females, respectively, which represented the 10th and 90th centiles in Ines charts). Informed consent was obtained from the mothers of all subjects donating umbilical cords following the Declaration of Helsinki.

### 2.2. Cell Isolation and Characterization

Primary female HUVECs (FHUVECs) and male HUVECs (MHUVECs) were isolated using collagenase treatment (Sigma-Aldrich, Milano, Italy), as previously described by Addis et al. [55], and cultured in plates pre-coated with 1% gelatine (Sigma-Aldrich, Milano, Italy) in M199 medium (Life Technologies, Monza, Italy) supplemented with 10% fetal bovine serum (FBS) (Life Technologies, Monza, Italy), 10% new born calf serum (NBCS) (Life Technologies, Monza, Italy), 1% antibiotic/antimycotic (Sigma-Aldrich, Milano, Italy), and 2 mM of l-glutamine (Sigma-Aldrich, Milano, Italy) until confluence in a 5% CO_2_ humidified atmosphere.

As previously described [55], cultured cells were characterized as endothelial cells using the exhibition of cobblestone morphology when they were contact-inhibited and an evaluation of the expression of the von Willebrand factor, which is a glycoprotein that is constitutively stored in intra-endothelial Weibel–Palade granules.

FHUVECs and MHUVECs were used at passage 3 to ensure their endothelial characteristics, and all experiments were conducted in duplicate or triplicate. Twenty-four hours before experiments, 50,000 cells at P3 for each experimental condition were suspended in M199 medium without phenol red (Life Technologies, Monza, Italy) and supplemented with 5% FBS and 5% new born calf serum (NBCS) (Life Technologies, Monza, Italy), 1% antibiotic/antimycotic (Sigma-Aldrich, Milano, Italy), and 2 mM of l-glutamine (Sigma-Aldrich, Milano, Italy) to minimize the potential effect of sex hormones contained in the bovine serum.

### 2.3. Experimental Procedures

The experimental groups were: a) non-irradiated HUVECs (basal cells, 10 mM TAU- and 5 mM NAC –pre--treated cells) and b) irradiated HUVECs (untreated cells irradiated with 1.6 and 3.2 Gy X-rays; 10 mM TAU-pre-treated and 1.6 and 3.2 Gy irradiated cells; and 5 mM NAC-pre-treated and 1.6 and 3.2 Gy irradiated cells).

Cells were pre-treated with TAU 10 mM or NAC 5 mM (Sigma-Aldrich, Milano, Italy) 24 h before irradiation. Concentrations of the antioxidants were chosen based on the data available in the literature on HUVECs [56,57,58,59,60]. Untreated and pre-treated cells were irradiated. The irradiation was performed through an X-ray tube working at 80 kV and 5 mA (Gilardoni S.p.A, Italy). A Plexiglas layer 1 cm thick filtered the low energy part of radiation. The dose rate of about 0.2 Gy/min was continuously monitored by a DAP camera (Dose Area Product, VacuDAP by VacuTEC, Germany) placed together with the cell holder (microvials or multiwells). The following X-ray doses were used for the experiments: 1.6, 3.2, 6, and 12 Gy. At the highest doses (6 and 12 Gy), the decrease in the viability was higher than 50%, and the increase in LDH release was about 80% in male and female HUVECs. Therefore, these doses were not used for subsequent analysis.

After irradiation, the cells from each vial were seeded in a 96 well for each experimental condition (about 15,000 cells/well in triplicate). Crystal violet assay and LDH release were used to assess cell viability and cytotoxicity 24 h after the seeding. Basal cells were subjected to the same experimental conditions, except for irradiation and pre-treatments.

### 2.4. Cell Viability

Cell viability was determined using the crystal violet assay according to [61]. The absorbance was recorded at 540 nm, and the percentage of viability was calculated compared with basal cells, for which a value of vitality equal to 100% was assumed.

### 2.5. LDH Assay

LDL release was measured in a culture medium from irradiated and non-irradiated cells pre-treated or not treated with TAU and NAC using the LDH Cytotoxicity Detection kit (Roche Diagnostics, Monza, Italy) and following the manufacturer’s instructions. LDL release was expressed as the percentage of the LDH measured in the medium divided by the LDH release measured after cell treatment with 2% Triton X-100 (positive control, 100% LDH release).

### 2.6. Wound Healing Assay

Cells were grown to confluence in gelatine-coated 12-well plates in a complete medium. Confluent cells were manually scratched in each well using a p10 pipette tip, and the cells were cultured for 48 h. Photographs were taken just after scratching and after 6, 9, 24, and 48 h of incubation at a × 4 magnification. The percentage of wound closure was calculated using ImageProPlus software (Media Cybernetics, Inc, Rockville, MD, USA) by measuring the wound area at each time point compared with the initial area measured at the time of the scratch. Each sample was assayed in duplicate.

### 2.7. MDA Determination

MDA was determined as previously described [62] using 10 μg of cell lysates. The quantification was performed spectrophotometrically at 535 nm by measuring the absorbance produced by 100 μL of the sample. Calibration curves were built with standards of MDA at 5, 10, 25, and 50 μM. Each sample was assayed in duplicate.

### 2.8. Western Blotting

The protein concentration was quantified using the BCA protein assay kit (Thermo Scientific, Waltham, MA, USA). For the Western blot analysis, 25 μg of solubilized protein was electrophoretically resolved by 4–15% SDS-PAGE (100 V, 2 h, 24 °C) and then transferred to a PVDF membrane (250 mA, 65 min, 4 °C) using a Transblot-turbo system (Bio-Rad, Milano, Italy). The membranes were blocked in 5% (w/v) skim milk (Sigma-Aldrich, Milano, Italy) in 150 mM Tris buffer (Sigma-Aldrich, Milano, Italy) and 20 mM Tris-HCl, pH 7.2 (Sigma-Aldrich, Milano, Italy) at 24 °C for 1 h and then incubated overnight at 4 °C with the following antibodies, all produced in rabbit and diluted 1:1000: α-actin (Sigma-Aldrich, Milano, Italy), LC3 (MBL, Milano, Italy), and caspase-9 (Cell Signaling Technology, Milano, Italy). After washing, the blots were incubated for 1 h with horseradish peroxidase (HRP)-conjugated secondary antibody (Cell Signaling Technology, Milano, Italy) (1:2000). Antibody binding was detected using a chemiluminescence reaction (Cell Signalling Technology, Danvers, MA, USA) with the Bio-Rad Chemi Doc instrument (Berkeley, CA, USA). Band volume analysis was performed using Image Lab 4.0 software (Bio-Rad Laboratories, Berkeley, CA, USA), and densitometric data were normalized based on α-actin levels, which did not differ in MHUVECs and FHUVECs [55].

### 2.9. Statistical Analysis

Data were reported as the mean ± standard deviation (SD). Statistical analysis was performed using Two Way Analysis of Variance followed by the Pairwise Multiple Comparison Procedures to analyze the effect of sex, X-rays, and treatments using Sigma-Stat 3.1 software (Systat Software, Erkrath, Germany). The distribution of samples was assessed via the Kolmogorov–Smirnov and Shapiro tests.

Linear regression analysis was performed by plotting time against the percentage of wound closure and comparing slope variations through a global test of coincidence using Sigma-Stat 3.1 software (Systat Software, Erkrath, Germany). A *p* ≤ 0.05 was considered statistically significant.

## 3. Results

### 3.1. Characteristics of Donors

The mothers of female and male neonates did not differ significantly in age and body weight, and neonates of both sexes did not diverge significantly in body weight (Table 1).

### 3.2. Effect of X-rays on HUVECs Viability and Lactate Dehydrogenase (LDH) Release

**Figure 1:**
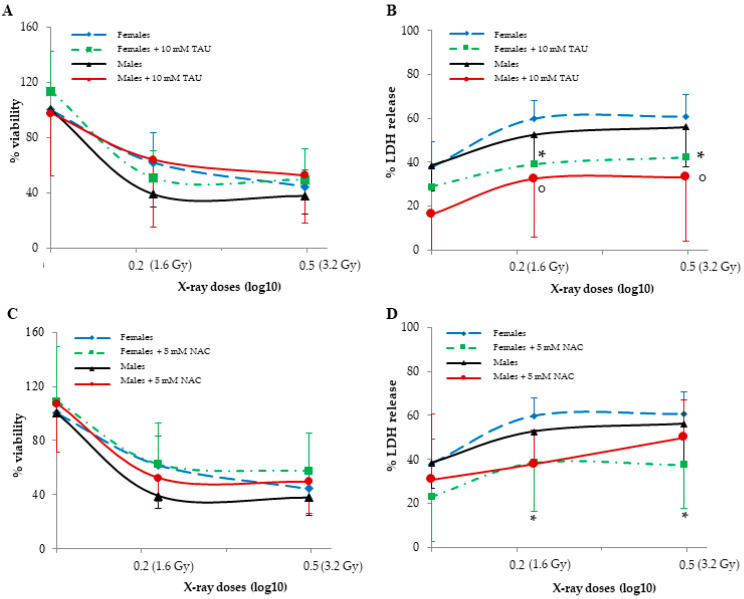
The effects of TAU and NAC pre-treatments on viability (**A**,**C**) and LDH release (**B**,**D**) in non-irradiated and irradiated female and male HUVECs. Data are reported as the means ± SD of 6–7 samples for each sex and dose. ° represents a *p* < 0.05 versus non-irradiated cells in MHUVECs, while * represents a *p* < 0.05 versus non-irradiated cells in FHUVECs.

Viability and LDH release in basal cells, a measure of cytotoxicity, did not present sexual dimorphism. The irradiation of cells with X-rays at doses of 1.6 and 3.2 Gy reduced cell viability in a statistically significant manner, but this occurred regardless of cell sex (Figure 1A,C), and irradiation increased LDH release in a dose-dependent manner, regardless of cell sex (Figure 1B,D).

### 3.3. Effect of X-rays on HUVECs Migration

**Figure 2:**
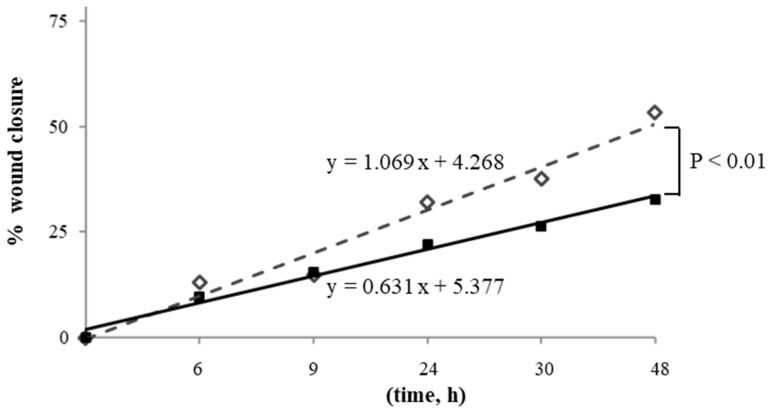
Linear regression analysis of cell migration in MHUVECs (■) and FHUVECs (◊) after exposure to 3.2 Gy of X-rays.

Migration, expressed as the percentage of wound closure that did not diverge between the sexes, and closure were completed in 48 h in basal cells. X-rays, instead, significantly reduced wound closure in both MHUVECs and FHUVECs (Table 2). In detail, at 1.6 Gy, both male and female cells showed a slower and partial recovery compared with basal cells and had no significant sex difference (Table 2), which was also confirmed by linear regression analysis that showed similar slopes when 1.6 Gy irradiated MHUVEC were compared with FHUVECs (y = 1.351x + 0.784 and y = 1.454x + 2.131 for MHUVECs and FHUVECs, respectively). A 3.2 Gy X-ray produced a longer delay in wound closure than 1.6 Gy, and its effects were associated with sex, which was also confirmed by linear regression analysis which showed that the slopes significantly diverged between 3.2 Gy-irradiated MHUVECs and 3.2 Gy-irradiated FHUVECs (Figure 2), suggesting that wound repair was more rapid in FHUVECs than in MHUVECs.

### 3.4. Effect of X-rays on Autophagy

**Figure 3:**
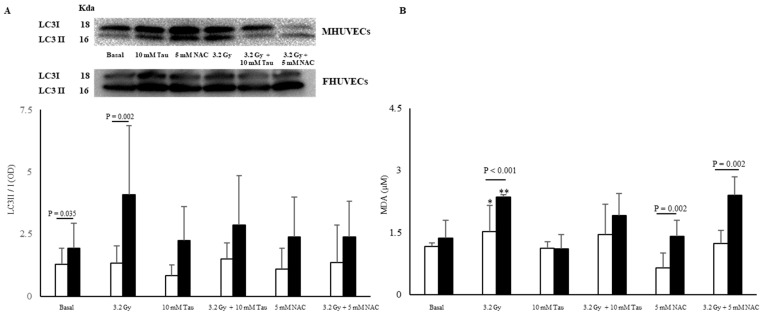
LC3II/I ratio (**A**) and MDA levels (**B**) in non-irradiated and irradiated MHUVECs (white bars) and FHUVECs (black bars) before and after pre-treatments. Data are reported as the means ± SD of at least 3 independent experiments. * *p* = 0.032 basal vs. 3.2 Gy in MHUVECs; ** *p* < 0.001 basal vs. 3.2 Gy in FHUVECs.

Autophagy is a catabolic process that delivers cellular constituents, including damaged or superfluous organelles and long-lived proteins, to lysosomes for degradation and recycling [21]. In basal conditions, it was measured via a LC3II/I ratio and was significantly higher in FHUVECs. After 3.2 Gy irradiation, the LC3II/I ratio significantly increased (about 110%) only in FHUVECs (Figure 3A), while it was similar to those of basal cells in irradiated MHUVECs.

### 3.5. Effect of X-rays on Lipid Peroxidation

Malondialdehyde (MDA) levels did not significantly diverge in basal male and female HUVECs, but they significantly increased in the irradiated cells of both sexes. In particular, the increase was significantly more pronounced in FHUVECs than in MHUVECs (Figure 3B).

### 3.6. Effect of Pre-Treatments on HUVECs Viability and LDH Release

In basal FHUVECs and MHUVECs, 10 mM TAU and 5 mM NAC did not affect viability and LDH release (Figure 1A,B), whereas they reduced LDH release in irradiated cells (Figure 1C,D). In detail, 10 mM TAU and 5 mM NAC had both a significant protective effect in LDH reduction in FHUVECs, and TAU was more protective in male cells than NAC (Figure 1C,D). In fact, after irradiation, TAU reduced LDH release in MHUVECs, while NAC had no effect.

### 3.7. Effect of Pre-Treatments on HUVECs Migration

**Figure 4:**
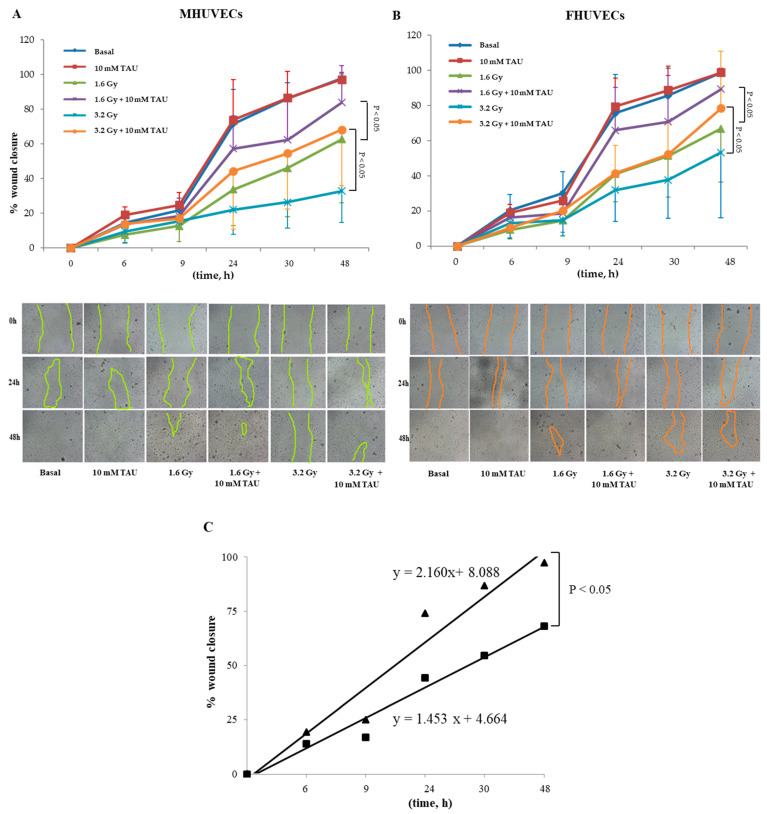
(**A**,**B**) The effect of 10 mM TAU on the migration of irradiated and not irradiated MHUVECs and FHUVECs (data are means ± SD of 5 independent experiments for each sex and dose). (**C**) Linear regression analysis of data illustrated in A e B of migration in 3.2 Gy-irradiated MHUVECs pre-treated with TAU (▄) and non-irradiated MHUVECs pre-treated with TAU (▲).

TAU and NAC did not modify the migration of non-irradiated HUVECs for both sexes (Figure 4A,B). However, TAU significantly promoted the closure of the wound in 1.6 and 3.6 Gy irradiated cells, especially in the late phase (48 h), but this occurred independently of cell sex (Figure 4A,B).

Moreover, linear regression analysis showed that the slopes significantly diverged between 3.2 Gy irradiated and non-irradiated TAU-pretreated MHUVECs (Figure 4C). No other statistically significant differences emerged from the linear regression analysis.

**Figure 5:**
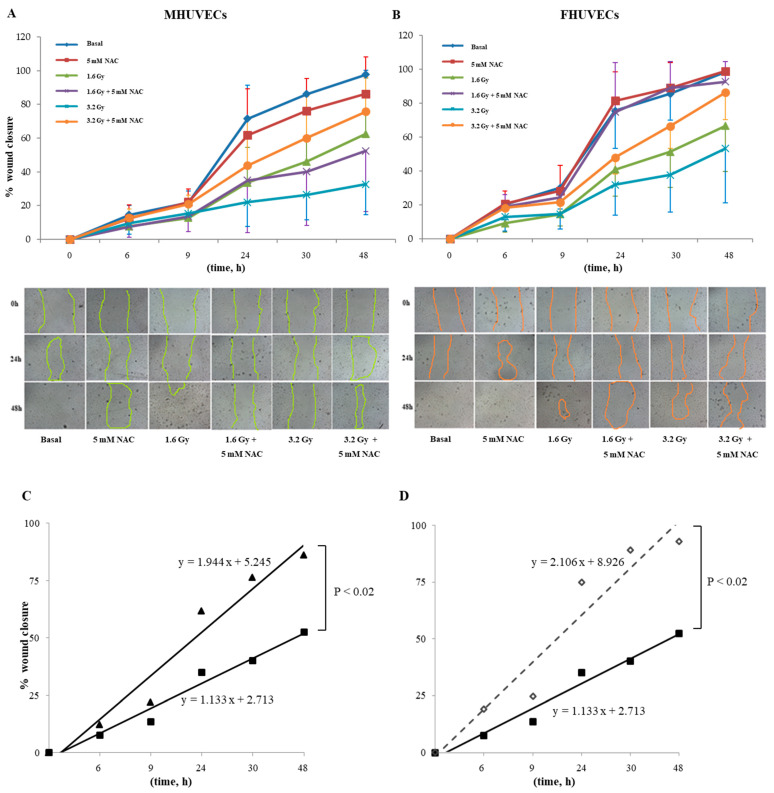
(**A**,**B**) The effect of 5 mM NAC on the migration of MHUVECs and FHUVECs. (**C**,**D**) Linear regression analysis of data illustrated in A e B on the migration of 1.6 Gy-irradiated pre-treated with NAC in MHUVECs (■) and FHUVECs (◊). (**D**) Linear regression analysis of data illustrated in B of the migration of 1.6 Gy-irradiated MHUVECs pre-treated with NAC (■) and non-irradiated MHUVECs pre-treated with NAC (▲).

NAC pre-incubation did not affect male and female HUVECs migration when compared with basal cells (Figure 5A,B). The regression analysis evidenced a significant difference in slopes between NAC-irradiated MHUVECs versus non-irradiated NAC-pretreated MHUVECs (Figure 5C), which indicated a positive effect of NAC on cellular migration.

Moreover, linear regression analysis showed that slopes significantly diverged between MHUVECs and FHUVECs pretreated with NAC and exposed to 1.6 Gy (Figure 5D), which indicated that NAC is more effective in FHUVECs. No other statistically significant differences emerged from the linear regression analysis.

### 3.8. Effect of Pre-Treatments on Autophagy

In non-irradiated male and female cells, 10 mM TAU and 5 NAC pre-incubation did not significantly affect autophagy (expressed as the LC3II/I ratio), but in irradiated FHUVECs, they attenuated the autophagy in a non-statistically significant manner as they were practically inactive in irradiated MHUVECs (Figure 3A).

### 3.9. Effect of Pre-Treatments on Lipid Peroxidation

Ten mM TAU pre-incubation did not significantly affect lipid peroxidation in both non-irradiated and irradiated MHUVECs and FHUVECs compared with basal cells (Figure 3B), although a non-significant reduction in MDA was observed in FHUVECs.

However, NAC pre-incubation highlighted sex differences in the lipid peroxidation as MDA levels were statistically significantly higher in FHUVECs than in MHUVECs (Figure 3B).

## 4. Discussion

The biological and molecular mechanisms underlying IR damage are still not fully understood [19], and it is even less known whether IR damage is influenced by sex. In this study, we show that viability, LDH release, cell migration, and lipid peroxidation do not vary between sexes in basal conditions, while autophagy is higher in female cells than in male ones. Globally, this indicates that sex differences are parameter specific, which has already been shown in other experimental models [55,63,64]. Indeed, the data regarding autophagy are not in line with previous results [55], but the discrepancy probably depends on differences in serum concentrations in the culture medium.

Sexual polymorphism is also related to cell migration and MDA levels in irradiated cells, whereas changes in cell viability and cytotoxicity are sex-independent. These findings indicate that IR amplifies sex differences in a parameter specific manner. Radiation-induced autophagy may have a different role in cell fate depending on the dose and duration of radiation leading to survival or death [65]. As a close link between oxidative stress and autophagy was described [63,66], the observed increase in lipid peroxidation could explain, at least in part, the increase in autophagy observed in irradiated cells. However, it does not explain the results obtained for the cells preincubated with NAC where both irradiated and non-irradiated lipid peroxidation are higher in female cells, but the autophagy is scarcely affected. Other sex differences after irradiation have been described in human male lymphocytes, which are less sensitive than female cells when exposed to 30 Gy X-rays [67], but other studies do not observe any significant sex differences in human hematopoietic stem cells irradiated with X-rays (0.5 and 2 Gy) [68]. This suggests that sex differences are related to the cell type, the radiation dose, and the studied parameter.

The development of non-toxic agents to combat radiation-induced endothelial dysfunction is of paramount importance because alterations in endothelial function affect the control of vascular tone, angiogenesis, hemostasis, inflammation, vascular integrity, and vessel repairing and the provision of an antioxidant, anti-inflammatory, and antithrombotic interface [69]. Some of these processes appear to be sex-dependent [70]. In non-irradiated cells, 10 mM TAU does not modify any of the studied parameters compared with basal cells, except for the LC3II/I ratio. In fact, TAU attenuates the sex difference in the autophagic response. In irradiated cells, TAU reduces cytotoxicity in male and female cells, and is ineffective regarding viability. In addition, it promotes cell migration after radiation at 24 and 48 h and decreases the autophagic process expressed as the LC3II/I ratio. It does not affect MDA levels. In particular, TAU increases the migration capacity, especially in the late phases in both sexes. TAU prevents apoptosis induced by hyperglycemia [57,71], lipopolysaccharide, and tumor necrosis factor-alpha stimulation and reduces oxidative stress [72] in HUVECs and other human endothelial cells not stratified for sex. In vitro, it declines high potassium-induced contraction in rabbit ear arteries [73]. Furthermore, TAU administered in vivo attenuates low-density lipoprotein-induced endothelial dysfunction [74]. Globally, the above data also suggest that the activity of TAU is target-specific, and its effect may be due to a combination of different mechanisms as proposed by Christophersen [36], although the author does not focus on the sex effect. The small beneficial effects observed regarding TAU could be of great relevance in cancer irradiated patients who appear to be TAU-depleted after cytotoxic chemotherapy and/or radiotherapy [37].

Pre-treatment with the glutathione precursor NAC [29,40] reduces cytotoxicity in male and female cells and promotes wound closure at 24 h and 48 h after radiation, particularly in FHUVECs. Moreover, NAC cancels sex differences in autophagy in irradiated and non-irradiated cells. Finally, in both irradiated and non-irradiated cells, NAC exposure brings out a sex difference in MDA levels, which are higher in females. Millimolar NAC leads to a higher rate of wound closure than the controls 36 h after wounding in human skin fibroblast cell lines not stratified for donor sex [75]. The sex differences observed with NAC are not surprising because glutathione metabolism shows significant sex differences [10]; for example, intracellular glutathione synthesis requires glutamate-cysteine ligase, which is less expressed in the female liver than in the male liver, at least in rats [62].

Globally, our data show some small protective and sex-specific effects of TAU and NAC. In particular, both promote a decrease in X-ray-mediated cytotoxicity. TAU is more effective in promoting wound closure in MHUVECs, while NAC is more effective in FHUVECs. Moreover, TAU does not affect autophagy, while NAC attenuated the differences between the sexes observed in the autophagic response.

Finally, a sex-specific effect of NAC on MDA levels can be noted as it increases levels for females. On the contrary, TAU does not modify this parameter. Overall, these data suggest that the two antioxidants may mediate sex-specific protective effects through different mechanisms, although the effect of NAC seems to be more influenced by sex, and this aspect could be in line with sex differences described in glutathione metabolism and glutathione cycle [10].

In conclusion, TAU and NAC have similar safety and tolerability in non- irradiated MHUVECs, while NAC is less safe than TAU in non-irradiated FHUVECs because it increases lipid peroxidation. Cell irradiation increases autophagy only in FHUVECs where it produces a more marked elevation in MDA and a more rapid wound closure than in MHUVECs. In irradiated cells, NAC preincubation has a positive effect on cellular migration and LDH release, which is more effective in FHUVECs. However, TAU significantly promoted the closure of the wound and a decrease in LDH release independently of cell sex in the same experimental conditions. Thus, taurine appears to be more protective than NAC in male cells.

A further understanding of radiation-induced endothelial dysfunction could lead to progress in the development of countermeasures, such as antioxidant or mitigator therapies, for cardiovascular diseases in subjects exposed to radiation.

Finally, our results confirm and stress the importance of reporting cell sex in experiments and including the sex-gender variable in preclinical and clinical research [2] to understand sex-specific mechanisms and create personalized diagnostic and therapeutic approaches. Moreover, these results allow us to lay the groundwork for a sex-specific use of antioxidants.

## Figures and Tables

**Table 1:** Physical data of the enrolled cohorts.

	Age of Mothers (Years)	Body Weight of Mothers (kg)	Body Weight of Neonates (kg)
**Males (*n* = 9)**	31.2 ± 5.9	65.3 ± 7.0	3.3 ± 0.3
**Females (*n* = 10)**	30.6 ± 4.4	62.3 ± 6.4	3.3 ± 0.4

Values are reported as the mean ± SD.

**Table 2:** The effect of X-rays on HUVECs migration.

		6 h	9 h	24 h	30 h	48 h
**Basal**	MHUVECs	14.6 ± 5.7	21.8 ± 6.8	71.5 ± 19.8	86.1 ± 9.2	97.7 ± 2.6
	FHUVECs	20.2 ± 8.9	30.1 ± 12.3	75.6 ± 22.1	85.6 ± 15.7	98.5 ± 1.9
**1.6 Gy**	MHUVECs	7.7 ± 4.8 °	12.7 ± 8.9	33.6 ± 20.8 °	46.1 ± 28.1 °	62.6 ± 36.6
	FHUVECs	9.2 ± 5.2 *	14.6 ± 6.8 *	41.0 ± 15.9 *	51.4 ± 23.6 *	66.7 ± 30.2
**3.2 Gy**	MHUVECs	9.5 ± 6.4	15.4 ± 3.9	22.1 ± 14.3 °	26.4 ± 14.8 °	32.7 ± 17.9 °
	FHUVECs	13.0 ± 8.3	14.8 ± 8.9 *	31.9 ± 18.0 *	37.7 ± 21.9 *	53.3 ± 37.1 *

Data are reported as the means ± SD of the percentage of wound closure in FHUVECs and MHUVECs of 5 independent experiments performed in duplicate (for each sex and dose). ° represents a *p* < 0.05 versus non-irradiated cells in MHUVECs, while * represents a *p* < 0.05 versus non-irradiated cells in FHUVECs.

## Data Availability

Data will be made available on request.
